# Beyond Fasting Lipids: Nutritional and Clinical Perspectives on Postprandial Triglycerides

**DOI:** 10.3390/nu18081222

**Published:** 2026-04-13

**Authors:** Oana Patru, Andrei Paunescu, Bogdan Enache, Silvia Luca, Cristina Vacarescu, Andreea-Iulia Ciornei, Dragos Cozma, Andreea Bena, Constantin-Tudor Luca, Simina Crisan

**Affiliations:** 1 Cardiology Department, “Victor Babes” University of Medicine and Pharmacy, 2 Eftimie Murgu Sq., 300041 Timisoara, Romania; oana.patru@umft.ro (O.P.); silvia.luca@umft.ro (S.L.); cristina.vacarescu@umft.ro (C.V.); dragos.cozma@umft.ro (D.C.); constantin.luca@umft.ro (C.-T.L.); simina.crisan@umft.ro (S.C.); 2 Research Center of the Institute of Cardiovascular Diseases Timisoara, 13A Gheorghe Adam Street, 300310 Timisoara, Romania; 3 Doctoral School, “Victor Babes” University of Medicine and Pharmacy, 300041 Timisoara, Romania; andreea.ciornei@umft.ro; 4 Department of Urology, “Pius Brînzeu” Emergency Clinical County University Hospital, 300723 Timisoara, Romania; ai.paunescu@gmail.com; 5 Institute of Cardiovascular Diseases Timisoara, 13A Gheorghe Adam Street, 300310 Timisoara, Romania; 6 Discipline of Endocrinology, Second Department of Internal Medicine, “Victor Babes” University of Medicine and Pharmacy, 300041 Timisoara, Romania; bena.andreea@umft.ro; 7 Center for Molecular Research in Nephrology and Vascular Disease, “Victor Babes” University of Medicine and Pharmacy, 300041 Timisoara, Romania

**Keywords:** postprandial triglycerides, lipid metabolism, triglyceride-rich lipoproteins, remnant cholesterol, non-fasting lipids, dietary patterns, metabolic flexibility, residual cardiovascular risk, cardiometabolic health

## Abstract

Background: Postprandial triglyceride (TG) metabolism represents a dynamic dimension of lipid physiology that complements conventional fasting lipid assessment. Although low-density lipoprotein cholesterol (LDL-C) remains the primary therapeutic target in cardiovascular prevention, residual cardiovascular risk persists in many individuals despite apparently adequate fasting lipid control. Because most individuals spend the majority of their waking hours in a fed state, postprandial TG responses may provide clinically relevant insight into metabolic flexibility, dietary exposure, and the efficiency of TG-rich lipoprotein clearance. Methods: This narrative review was conducted using a literature search guided by predefined themes, keywords, and databases, without following a formal systematic review protocol. Randomized controlled trials, observational studies, meta-analyses, and major reviews addressing postprandial lipid metabolism, dietary determinants, and cardiometabolic risk were included, with priority given to human studies. Results: Postprandial TG responses are strongly influenced by dietary composition, eating patterns, and metabolic health. Individuals with insulin resistance, type 2 diabetes, obesity, and metabolic-associated steatotic liver disease (MASLD) frequently demonstrate exaggerated or prolonged postprandial lipemia even when fasting TG concentrations appear acceptable. While circulating TGs serve as practical clinical markers of postprandial lipid handling, cholesterol-enriched remnant lipoproteins more closely reflect atherogenic burden. Nutritional interventions, weight management, and physical activity consistently improve postprandial TG dynamics, whereas pharmacologic therapy provides additional benefit in selected high-risk patients. Non-fasting TG measurements may provide additional insight into postprandial lipid metabolism and residual cardiovascular risk, although standardized protocols and validated clinical thresholds remain to be established. Conclusions: Postprandial TG metabolism provides clinically meaningful information beyond fasting lipid measurements and represents a useful adjunct for refining residual cardiovascular risk assessment. Although standardized protocols remain limited, integrating nutritional and clinical perspectives may support a more comprehensive and individualized approach to cardiometabolic prevention.

## 1. Introduction

Cardiovascular diseases remain the leading cause of mortality worldwide despite substantial advances in prevention and treatment [1]. Lowering low-density lipoprotein cholesterol (LDL-C) through dietary and pharmacological interventions has consistently reduced cardiovascular events; however, a considerable proportion of myocardial infarctions and strokes continues to occur even among individuals achieving guideline-recommended LDL-C targets. This persistent burden has led to increasing recognition of residual cardiovascular risk, reflecting the multifactorial nature of atherosclerosis in which lipid fractions beyond LDL-C, together with metabolic and lifestyle-related factors, contribute to ongoing vascular injury. Thus, LDL-C reduction, while essential, does not eliminate cardiovascular risk [2,3,4,5].

Among these additional factors, triglycerides (TGs) and triglyceride-rich lipoproteins (TRLs) have received renewed attention. Elevated TG concentrations are consistently associated with increased cardiovascular risk, particularly in individuals with insulin resistance, obesity, or type 2 diabetes [6,7]. However, this relationship is not necessarily causal per se, as growing evidence indicates that the atherogenic risk is more directly mediated by TRLs and their remnants, especially remnant cholesterol. Traditionally, lipid assessment has relied on fasting measurements, largely for methodological convenience rather than physiological relevance. In everyday life, individuals spend most waking hours in a fed or postprandial state, and several professional societies now consider non-fasting lipid profiles acceptable for routine cardiovascular risk screening [8,9,10]. This shift has renewed interest in TG fluctuations after meals and their clinical significance.

The postprandial state is characterized by intestinal production of chylomicrons and hepatic secretion of very-low-density lipoproteins (VLDLs), leading to transient elevations in circulating TGs and remnant particles. In metabolically healthy individuals, TGs typically peak 3–4 h after a mixed meal and return toward baseline within several hours, whereas insulin resistance, central adiposity, or hepatic steatosis are associated with higher peaks and delayed clearance. TGs themselves are not directly deposited within the arterial wall; rather, they are transported within TRLs, which undergo progressive lipolysis, generating smaller remnant particles enriched in cholesterol. These remnant lipoproteins can penetrate the arterial intima and contribute directly to atherogenesis. This mechanism supports the concept that TGs act primarily as markers of atherogenic remnant lipoprotein burden rather than direct causal agents [11,12,13]. Standardized cut-off values for defining abnormal postprandial TG responses remain lacking. However, several studies and expert consensus statements have proposed indicative non-fasting thresholds, most commonly in the range of approximately 175–220 mg/dL (≈2.0–2.5 mmol/L). These values vary depending on study design, timing of measurement, and population characteristics, and should therefore be interpreted with caution in the absence of universally accepted protocols [10,14]. In this context, the present review does not aim to define specific thresholds, but rather to synthesize current evidence and highlight the methodological and biological factors that currently limit standardization while outlining key considerations for future research.

Nutrition plays a central role in shaping postprandial TG metabolism. Meal composition, dietary fat quality, carbohydrate characteristics, alcohol intake, and eating patterns, including meal timing and frequency, influence the magnitude and duration of TG excursions. However, nutritional counseling in cardiovascular prevention continues to focus primarily on fasting lipid values, while the acute metabolic consequences of meals receive comparatively little attention. This gap is particularly relevant in contemporary dietary environments characterized by energy-dense foods, extended eating windows, and increasing metabolic disease prevalence [15,16,17].

Although TGs have been extensively studied, existing reviews often emphasize molecular mechanisms, epidemiological associations, or isolated dietary factors, with limited integration into a clinically oriented and nutrition-focused framework [18,19]. The aim of this narrative review is therefore to integrate current evidence on postprandial TG metabolism, discuss key nutritional and behavioral determinants, and explore their implications for cardiometabolic risk assessment and management. In particular, this review frames postprandial TG dynamics as a functional marker of metabolic flexibility and residual cardiovascular risk, bridging mechanistic insights with clinically relevant interpretation. In addition, the present work incorporates more recent evidence on metabolic dysfunction-associated steatotic liver disease (MASLD), ultra-processed food consumption, and circadian influences on lipid metabolism while also emphasizing the clinical relevance of non-fasting lipid assessment and remnant cholesterol. By integrating these elements with phenotype-specific nutritional and behavioral determinants, this review aims to provide a more comprehensive and clinically applicable perspective on postprandial TG metabolism.

**Figure 1:**
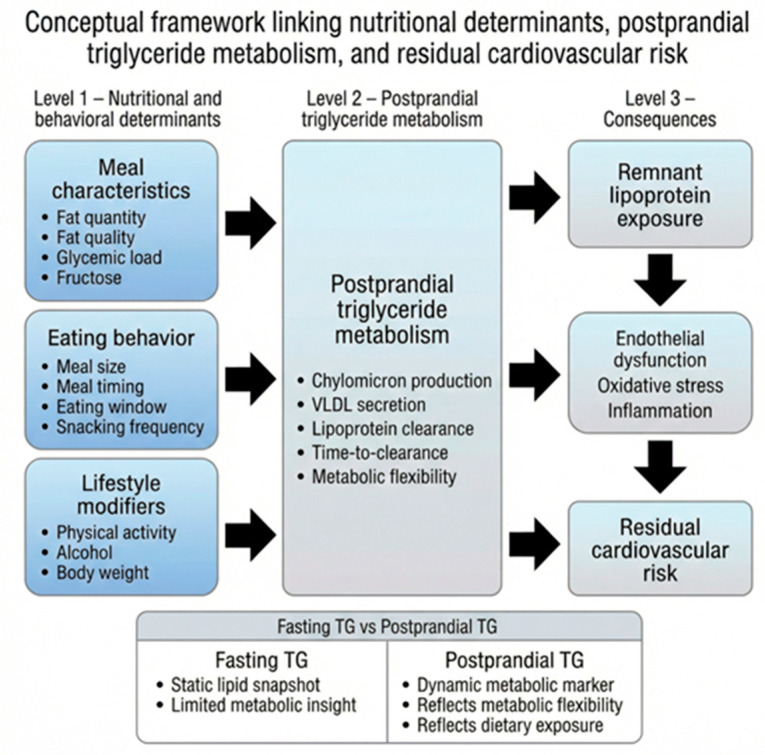
Conceptual framework linking nutritional determinants, postprandial TG metabolism, and residual cardiovascular risk. Created by the authors based on current literature.

The conceptual framework linking nutritional determinants, postprandial TG metabolism, and residual cardiovascular risk is illustrated in Figure 1 and provides the structural basis for the present review.

## 2. Materials and Methods

This narrative review was based on a literature search guided by predefined thematic areas related to postprandial triglyceride metabolism and its cardiovascular implications. The primary search was conducted in the PubMed and Scopus databases up to January 2026. The search strategy included combinations of relevant keywords such as “postprandial triglycerides”, “postprandial lipemia”, “triglyceride-rich lipoproteins”, “non-fasting triglycerides”, “diet and triglycerides”, “remnant cholesterol”, and “residual cardiovascular risk”, which were combined using Boolean operators (e.g., AND, OR) to refine the search. The search focused on publications from 2000 onwards, reflecting the evolution of contemporary concepts related to postprandial lipid metabolism, remnant cholesterol, and non-fasting lipid assessment. Earlier foundational studies were considered where necessary to support key mechanistic concepts. Additional relevant articles were identified through manual screening of reference lists from key reviews, consensus documents, and international clinical guidelines. Approximately 350 publications were screened for relevance, of which around 70–75 articles were considered central to the present synthesis. The screening process was conducted through title and abstract evaluation, followed by full-text assessment of potentially relevant articles, based on predefined thematic relevance and alignment with the objectives of the review. Article selection was based on relevance to the topic and contribution to the conceptual framework of the review. Eligible sources included randomized controlled trials, observational studies (prospective and cross-sectional), meta-analyses, consensus statements, and major narrative reviews addressing postprandial lipid metabolism, dietary determinants of triglyceride responses, and cardiometabolic risk. Priority was given to human studies reporting postprandial triglyceride dynamics, remnant lipoproteins, or cardiovascular and metabolic outcomes. Animal studies were included selectively when providing essential mechanistic insights with direct translational relevance. Publications not available in English, pediatric-only studies, case reports, and studies lacking clear clinical or mechanistic relevance were excluded.

Given the narrative nature of this review, no formal systematic risk-of-bias assessment or quantitative synthesis was performed. However, study selection and interpretation were guided by an implicit appraisal of methodological quality and level of evidence, with greater emphasis placed on randomized controlled trials, large prospective studies, and high-quality meta-analyses. Findings from lower levels of evidence were interpreted with appropriate caution. Efforts were made to balance foundational studies with recent high-impact publications in order to provide both historical context and an updated perspective on emerging concepts in postprandial lipid metabolism.

## 3. Postprandial TG Metabolism: Why It Matters Clinically

**Figure 2:**
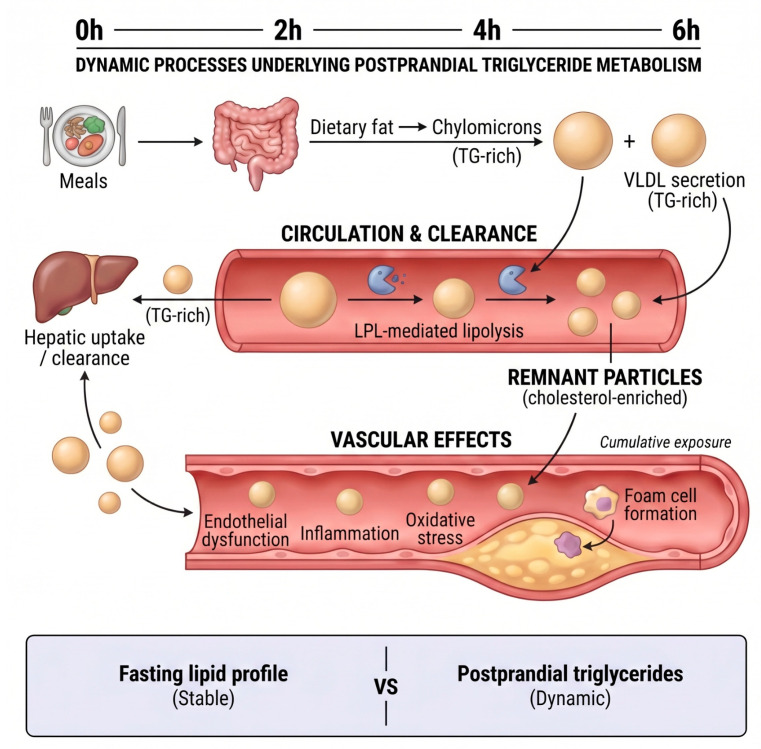
Dynamic processes underlying postprandial TG metabolism. Following a fat-containing meal, dietary TGs are incorporated into chylomicrons and released into the circulation, while the liver simultaneously secretes VLDL. These TRLs undergo lipoprotein lipase-mediated hydrolysis, generating smaller cholesterol-enriched remnant particles that are subsequently cleared by the liver. Delayed clearance results in prolonged vascular exposure to remnant lipoproteins, which may contribute to endothelial dysfunction, oxidative stress, inflammation, and foam cell formation. In contrast to relatively stable fasting lipid measurements, postprandial TGs reflect dynamic lipid metabolism in response to meals. Created by the authors based on current literature.

The postprandial state represents a metabolically dynamic period during which circulating TRLs increase in response to intestinal fat absorption and hepatic lipid secretion. Following a mixed meal containing fat, TGs are packaged into chylomicrons within enterocytes and released into the circulation, while the liver simultaneously secretes VLDL. This dual input produces a transient rise in plasma TG levels that typically peaks 3–4 h after food intake and gradually declines as TRLs are hydrolyzed by lipoprotein lipase and cleared as remnant particles [11,12,13,20,21]. In metabolically healthy adults, TG concentrations generally return toward baseline within several hours. In contrast, insulin resistance, central adiposity, or impaired hepatic lipid handling are associated with higher peaks and delayed clearance beyond 6–8 h, resulting in prolonged vascular exposure to TG-rich particles and their cholesterol-enriched remnants [14,22,23]. The dynamic processes underlying postprandial TG metabolism and vascular exposure to remnant lipoproteins are illustrated in Figure 2.

Remnant lipoproteins, generated from partially metabolized chylomicrons and VLDL, have attracted particular clinical interest because of their atherogenic potential. Unlike larger TG-rich particles, remnants are sufficiently small to penetrate the arterial intima, where they may be retained and taken up by macrophages, contributing to foam cell formation. Experimental, mechanistic, and epidemiological studies suggest that these particles are relatively enriched in cholesterol for their size, a feature often conceptualized as remnant cholesterol, and may promote endothelial dysfunction, oxidative stress, and local inflammatory signaling. Although TGs themselves are not directly deposited within atherosclerotic plaques, the lipoprotein carriers and their cholesterol-rich remnants represent biologically active entities capable of interacting with the vascular wall in ways that extend beyond lipid transport alone [24,25,26].

Postprandial lipid responses are also closely linked to inflammatory and endothelial pathways central to atherogenesis. Short-term elevations in TRLs have been associated in controlled feeding studies with transient increases in inflammatory mediators, impaired endothelium-dependent vasodilation, and enhanced oxidative stress [27]. These perturbations are generally modest and reversible in metabolically healthy individuals but become more pronounced and sustained in the presence of obesity, metabolic syndrome, or type 2 diabetes. This has led to the hypothesis that repeated daily postprandial TG excursions may contribute cumulatively to vascular exposure to atherogenic lipoproteins. This concept is supported primarily by observational associations and mechanistic studies, while direct longitudinal evidence remains limited. As such, this cumulative burden should be interpreted as a plausible pathophysiological framework rather than a definitively established causal mechanism [28,29,30,31].

From a clinical perspective, an important distinction exists between fasting LDL-C and postprandial TRLs. LDL-C reflects a relatively stable lipid fraction and remains a primary therapeutic target because of its well-established causal relationship with atherosclerotic cardiovascular disease. In contrast, postprandial TGs represent a dynamic marker that captures metabolic flexibility, dietary exposure, and the efficiency of lipoprotein clearance mechanisms. Individuals with optimal LDL-C values may still exhibit exaggerated postprandial TG responses, particularly in the presence of insulin resistance or hepatic steatosis, suggesting that fasting lipid panels alone may incompletely characterize cardiometabolic risk [10,14,32]. Rather than replacing LDL-C as a cornerstone of risk assessment, evaluation of postprandial TG metabolism provides complementary information that may help explain residual risk and identify individuals whose vascular exposure to atherogenic remnant lipoproteins remains underestimated in the fasting state.

Taken together, these observations support the view that postprandial TG metabolism may represent a clinically relevant dimension of lipid physiology that complements conventional fasting lipid assessment.

### Remnant Cholesterol and the Limits of TG as a Proxy

Circulating TG concentrations are widely used to characterize postprandial lipemia, yet they represent an indirect marker of lipoprotein metabolism rather than the atherogenic lipid burden itself. TGs primarily reflect the flux of lipid transport following dietary fat intake and hepatic VLDL secretion. In contrast, remnant cholesterol, defined as the cholesterol content carried within partially metabolized chylomicron and VLDL remnants, has emerged as a more proximal indicator of atherogenic potential. Unlike larger TRLs, remnants are sufficiently small to penetrate the arterial intima and are relatively enriched in cholesterol per particle, a characteristic that may amplify their contribution to foam cell formation, endothelial dysfunction, and vascular inflammation [10,14,33,34,35].

This distinction highlights an important conceptual limitation: elevated TG concentrations do not uniformly translate into elevated remnant cholesterol, and conversely, individuals with only moderate TG levels may still exhibit a disproportionate remnant burden. Postprandial TGs should therefore be interpreted less as a direct measure of dietary fat intake and more as a functional marker of metabolic flexibility and lipoprotein clearance efficiency. Within this framework, TG excursions serve as a practical clinical proxy for dynamic lipid handling, whereas remnant cholesterol more closely approximates the atherogenic component of postprandial lipoprotein metabolism. Recognizing this distinction helps explain why fasting TG values alone may underestimate residual cardiovascular risk and underscores the importance of contextual interpretation rather than reliance on isolated thresholds [9,34,36,37,38].

This conceptual perspective also clarifies why non-fasting TG measurements may provide functional insight into lipid clearance, whereas calculated or directly measured remnant cholesterol may better reflect cumulative atherogenic exposure. From a practical standpoint, remnant cholesterol can be estimated in routine clinical settings using standard lipid profiles, most commonly calculated as total cholesterol minus LDL-C minus HDL-C. While this approach offers a convenient surrogate, it remains influenced by fasting status and methodological variability, and direct measurement techniques are not yet widely standardized. Future clinical approaches may therefore benefit from integrating postprandial TG assessment with remnant cholesterol evaluation in order to better characterize residual cardiovascular risk, although further validation and standardization are required before routine implementation.

## 4. Dietary Determinants of Postprandial TGs

### 4.1. Macronutrient Composition

Meal macronutrient composition is a major determinant of the magnitude and duration of postprandial TG responses. Dietary fat quantity shows the most pronounced and consistent dose-dependent relationship with circulating TG excursions, with larger fat loads, often exceeding 30–40 g in controlled feeding trials, producing higher and more prolonged peaks. Beyond quantity, fat quality also influences postprandial lipid handling [21,39,40]. Randomized crossover studies indicate that meals enriched in saturated fatty acids are generally associated with less favorable TG clearance than those emphasizing mono- and polyunsaturated fatty acids, although individual variability and overall dietary context remain important modifiers. Mixed meals combining unsaturated fats, dietary fiber, and minimally processed ingredients have been associated with more efficient TG clearance and shorter lipemic duration [17,41,42,43].

Carbohydrate composition exerts an additional and often synergistic, but generally more variable, influence. High-glycemic-index carbohydrates and refined starches may amplify postprandial TG responses, particularly in individuals with insulin resistance or excess visceral adiposity, likely through increased hepatic very-low-density lipoprotein production and delayed lipid clearance [44,45,46]. Fructose intake is associated with increased hepatic de novo lipogenesis and higher circulating TG concentrations, especially when consumed as sugar-sweetened beverages or ultra-processed foods, whereas whole-food sources appear metabolically less disruptive [47,48,49,50,51]. In contrast, carbohydrate sources rich in dietary fiber and lower glycemic load tend to attenuate postprandial TG excursions, partly by slowing nutrient absorption and improving metabolic flexibility [51,52,53]. Adequate protein co-ingestion may exert a modest attenuating effect on postprandial glycemic and lipemic responses through effects on gastric emptying and insulin dynamics.

Overall, total dietary fat load appears to exert the strongest and most consistent effect on postprandial triglyceride responses, followed by fatty acid composition, while carbohydrate quality, protein co-ingestion, and overall meal structure demonstrate more variable and generally moderate effects depending on metabolic context. These effects are further amplified in individuals with insulin resistance or obesity, in whom postprandial lipid handling is consistently impaired [54,55].

Beyond individual macronutrients, dietary patterns provide a more integrative framework for understanding postprandial TG responses. Mediterranean-style dietary patterns, characterized by a high intake of monounsaturated fats, fiber, and minimally processed foods, have been consistently associated with more favorable postprandial lipemic profiles and improved remnant lipoprotein handling in both observational and interventional studies, including controlled trials such as CORDIOPREV. In contrast, Western dietary patterns rich in refined carbohydrates, saturated fats, and ultra-processed foods are more consistently associated with exaggerated and prolonged postprandial TG excursions and increased remnant lipoprotein exposure. Lower-carbohydrate and plant-based dietary patterns may also improve postprandial lipid responses, although their effects appear to depend on overall dietary composition and metabolic context. These observations highlight the relevance of dietary patterns as practical tools for modulating postprandial lipid metabolism beyond isolated nutrient effects [15,18,23,28,53].

### 4.2. Meal Pattern and Timing

Beyond meal composition, eating patterns strongly influence postprandial TG dynamics. Large or energy-dense mixed meals typically induce higher and more sustained TG elevations than smaller, evenly distributed meals, particularly in metabolically vulnerable individuals. Closely spaced eating occasions may result in cumulative lipemic exposure across the day, as TG concentrations often fail to return fully to baseline before subsequent meals. This pattern is increasingly relevant in modern dietary environments characterized by frequent snacking and extended daily eating windows [20,22,31,34,56,57].

Circadian factors and meal timing also interact with lipid metabolism. Human studies suggest that evening or late-night meals are associated with less efficient TG clearance and greater postprandial lipemic responses compared with earlier daytime intake. These observations may reflect diurnal variations in insulin sensitivity and hepatic lipid handling. Experimental and mechanistic studies further support a role for circadian regulation of lipid metabolism, although their direct translation to clinical practice remains limited. Although clear clinical thresholds remain undefined, structured meal timing and adequate metabolic recovery between meals appear to support more efficient lipid clearance [57,58,59,60].

### 4.3. Alcohol Intake and Ultra-Processed Food Context

Alcohol intake is a well-recognized modulator of TG metabolism, particularly in the postprandial state. Even moderate alcohol consumption with meals has been associated with delayed TG clearance and higher circulating concentrations in controlled feeding studies. These effects are partly attributed to increased hepatic TG synthesis and competition for oxidative pathways in the liver. The impact is more pronounced in individuals with pre-existing hypertriglyceridemia, insulin resistance, or hepatic steatosis [61,62,63,64,65].

Ultra-processed foods further exacerbate postprandial lipid disturbances through their characteristic combination of high energy density, refined carbohydrates, added sugars, industrial fats, and low fiber content. Such meals frequently deliver simultaneous glycemic and lipemic loads that challenge metabolic clearance mechanisms. Observational cohorts and short-term feeding trials have linked diets rich in ultra-processed foods with adverse cardiometabolic profiles, including elevated TG concentrations and reduced metabolic flexibility, sometimes independent of total caloric intake [66,67,68,69]. Overall, the broader dietary context and meal composition pattern, rather than isolated nutrients, appear to determine the cumulative postprandial TG burden.

**Figure 3:**
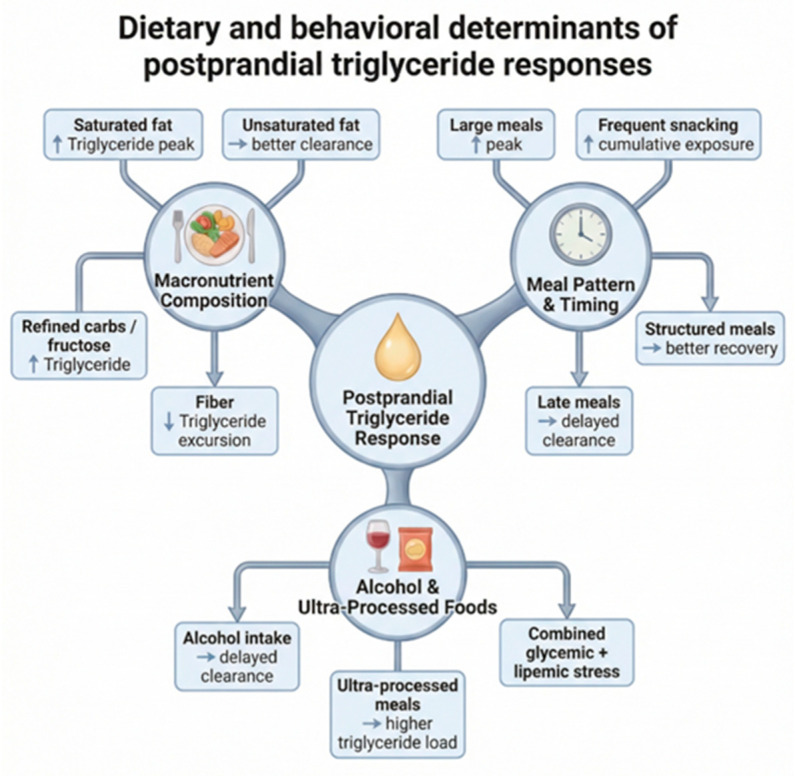
Dietary and behavioral determinants of postprandial TG responses. Postprandial TG responses are influenced by meal composition, eating patterns, and broader dietary context. Fat quantity and quality, carbohydrate characteristics, and dietary fiber intake modify the magnitude and duration of TG excursions. Meal size, meal timing, and eating frequency influence cumulative lipemic exposure and clearance efficiency. Alcohol intake and ultra-processed foods may further impair TG clearance and increase postprandial lipemic burden. Arrows indicate the general direction of association with postprandial TG responses. Created by the authors based on current literature.

The major dietary and behavioral determinants of postprandial TG responses are summarized in Figure 3.

## 5. Clinical Contexts Where Postprandial TGs Become Relevant

Postprandial TG responses are particularly informative in clinical settings characterized by impaired metabolic flexibility, where fasting lipid measurements may underestimate true vascular exposure to TRLs. Individuals with insulin resistance and type 2 diabetes consistently demonstrate exaggerated and prolonged postprandial lipemia compared with metabolically healthy controls, with controlled studies reporting higher TG area-under-the-curve and delayed return to baseline extending beyond the typical 6–8 h window. These alterations are attributed to increased hepatic VLDL secretion, reduced lipoprotein lipase activity, and delayed remnant clearance. Even in the presence of near-normal fasting TG values, postprandial excursions often remain elevated, suggesting that dynamic lipid handling captures additional aspects of metabolic dysfunction not fully reflected by fasting TG alone [4,7,25,28].

A similar pattern is observed in obesity and metabolic syndrome, conditions associated with central adiposity, chronic low-grade inflammation, and altered adipokine signaling. Excess visceral fat promotes increased free fatty acid flux to the liver and enhanced hepatic TG synthesis, contributing to elevated TG levels in both fasting and postprandial states. The postprandial period frequently reveals a disproportionate burden of cholesterol-enriched remnant lipoproteins that may not be evident in routine fasting lipid panels, supporting the value of postprandial assessment for refining cardiometabolic risk in individuals with increased adiposity [8,14,36,70,71,72].

MASLD represents another clinical context in which postprandial TG metabolism is commonly disturbed. Hepatic steatosis is associated with increased hepatic lipogenesis, impaired lipid oxidation, and altered very-low-density lipoprotein production, which may amplify TG excursions and prolong lipemic duration. Delayed clearance of TG-rich particles may coexist with only modest fasting TG elevations, suggesting that hepatic fat accumulation exerts an independent influence on postprandial lipid handling [73,74,75].

Additional contexts, although less extensively studied, also demonstrate clinically relevant alterations in postprandial TG metabolism. Women with polycystic ovary syndrome (PCOS) frequently exhibit insulin resistance-related dyslipidemia, including higher postprandial TG excursions [76]. Advancing age is associated with gradual declines in insulin sensitivity and lipid clearance efficiency, contributing to more sustained postprandial lipemia even in the absence of overt metabolic disease. Hormonal changes across the menopausal transition may further influence remnant lipoprotein dynamics [29,77].

Overall, postprandial TG responses vary substantially across populations and may serve as a sensitive marker of metabolic vulnerability. In clinical and research settings, non-fasting TG measurements are typically obtained within approximately 2–4 h after habitual food intake, reflecting a pragmatic approach to lipid assessment. However, the absence of standardized timing protocols remains a key limitation, as postprandial TG responses are influenced by meal composition, metabolic status, and time since ingestion [9,10]. In clinical practice, such variability may reveal discordance between apparently acceptable fasting lipid values and the actual daily burden of TG-rich and remnant lipoprotein exposure. A summary of key determinants influencing postprandial triglyceride responses and targeted nutritional strategies is presented in Table 1 (based on evidence from clinical trials, observational studies, and mechanistic research).

Taken together, these findings suggest that the relative importance of nutritional determinants, such as fat quality, carbohydrate composition, fructose intake, alcohol consumption, and meal timing, varies across clinical phenotypes, including insulin resistance/type 2 diabetes, obesity, MASLD, PCOS, and aging. This supports a more phenotype-specific approach to dietary modulation of postprandial TG responses.

## 6. Assessment in Clinical Practice

Assessment of TGs in routine clinical care has traditionally relied on fasting measurements; however, growing evidence supports the practicality and clinical relevance of non-fasting lipid testing in many situations. Because most individuals spend the majority of their waking hours in a fed state, non-fasting TG values may better reflect real-world metabolic exposure to TG-rich lipoproteins. Several professional societies now consider non-fasting lipid profiles acceptable for initial cardiovascular risk screening, particularly when TG levels are clearly within normal ranges. Non-fasting testing offers logistical advantages, improves patient compliance, and facilitates opportunistic screening, while markedly elevated values remain clinically meaningful irrespective of fasting status [9,10,11].

Interpretation becomes more nuanced when TG concentrations are borderline or moderately elevated. Intermediate non-fasting elevations may reflect recent dietary intake rather than persistent metabolic dysregulation, and confirmatory fasting measurements remain appropriate when TG levels exceed commonly accepted laboratory thresholds or when therapeutic decisions depend on precise lipid quantification. Rather than representing competing strategies, fasting and non-fasting measurements should be viewed as complementary tools, with the choice guided by clinical context and baseline metabolic risk.

Targeted evaluation of postprandial TG responses may be particularly informative in selected patient populations. Individuals with insulin resistance, type 2 diabetes, obesity, metabolic syndrome, or MASLD frequently exhibit exaggerated or prolonged postprandial lipemia even when fasting TG values appear acceptable. In such cases, a single fasting panel may underestimate exposure to TG-rich remnant particles and reveal discordance between fasting and non-fasting lipid profiles. Although formal oral fat tolerance tests remain largely confined to research settings, clinicians may still obtain practical insight from pragmatic approaches, such as measuring TGs several hours after a typical meal or evaluating trends across repeated measurements rather than relying on isolated values [9,10,11,14,78].

Important limitations currently restrict routine implementation of standardized postprandial testing. No universally accepted definition of abnormal postprandial TG response exists, and methodological heterogeneity across studies, particularly regarding meal composition, sampling intervals, and outcome metrics, limits translation into clinical practice. Laboratory reference ranges are typically derived from fasting populations, and substantial inter-individual variability related to diet, physical activity, circadian rhythm, and medication use further complicates interpretation [9,10,11,14,78]. Consequently, postprandial TG assessment should be regarded as an adjunctive rather than definitive diagnostic tool.

From a pragmatic clinical perspective, TG evaluation is best integrated into broader cardiometabolic assessment rather than considered in isolation. Non-fasting measurements are appropriate for general screening and follow-up in stable patients, whereas fasting confirmation is advisable when TGs are elevated, pancreatitis risk is a concern, or therapeutic thresholds are being considered. Ultimately, dynamic TG responses provide complementary insight into metabolic flexibility, remnant lipoprotein exposure, and residual cardiovascular risk, particularly in populations where fasting values alone may be misleading.

**Figure 4:**
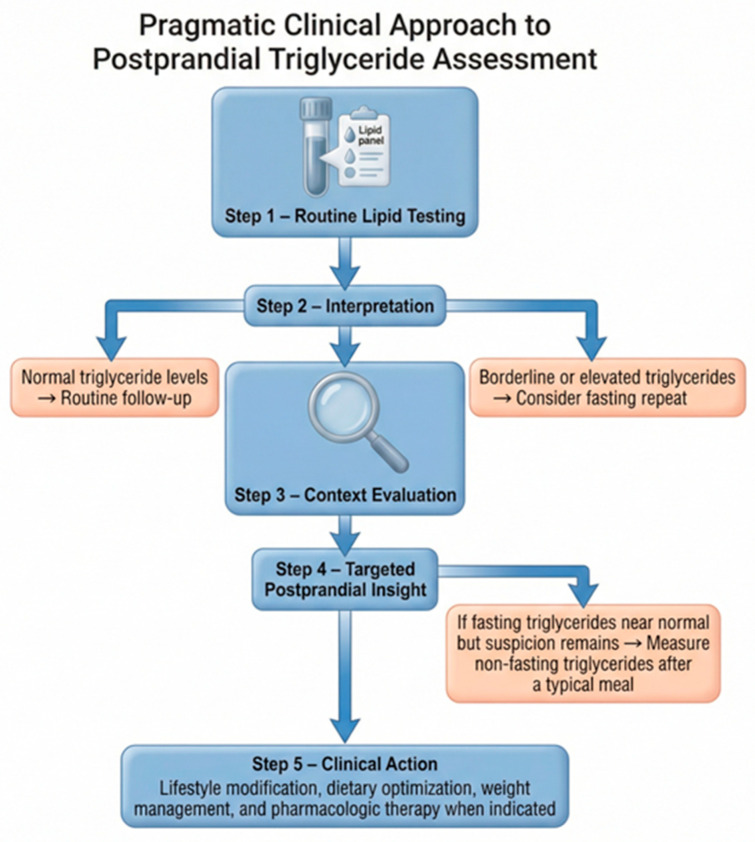
Pragmatic clinical approach to postprandial TG assessment. Routine lipid testing represents the first step in TG evaluation, followed by interpretation according to clinical context. Clearly normal TG values require routine follow-up, whereas borderline or elevated values may warrant confirmatory fasting measurements. In selected patients with suspected metabolic impairment despite near-normal fasting TGs, non-fasting measurements obtained after a typical meal may provide additional insight into postprandial lipid handling. Clinical management focuses on lifestyle modification, dietary optimization, weight management, and pharmacologic therapy when indicated. Created by the authors based on current literature.

A pragmatic clinical approach to postprandial TG assessment is illustrated in Figure 4.

## 7. Interventions: Nutritional and Pharmacological Perspectives

Management of exaggerated or prolonged postprandial TG responses requires an integrated approach in which lifestyle, nutrition, and pharmacologic therapy function as complementary rather than competing strategies. Because postprandial lipemia is strongly influenced by dietary composition, eating behavior, and overall metabolic health, non-pharmacological interventions represent the primary and most broadly applicable approach [8,18,79]. Pharmacologic therapies, when indicated, serve as an adjunct for patients with persistent hypertriglyceridemia or elevated cardiometabolic risk despite optimized lifestyle measures [19].

Dietary and lifestyle modification remains the cornerstone of intervention. Evidence from randomized feeding studies and longer-term behavioral interventions indicates that reducing excess energy intake, improving dietary fat quality, and limiting refined carbohydrates and added sugars are associated with more favorable postprandial TG dynamics. Dietary patterns emphasizing unsaturated fats, whole foods, and fiber-rich foods have been linked to improved TG clearance and reduced lipemic excursions. Weight reduction in individuals with overweight or obesity is commonly accompanied by improvements in insulin sensitivity and hepatic lipid handling, leading to attenuation of postprandial TG peaks. Both acute and habitual physical activity enhance TG clearance by increasing skeletal muscle lipoprotein lipase activity and improving metabolic flexibility. Structured meal patterns and avoidance of frequent energy-dense snacking may further reduce cumulative daily TG exposure. Collectively, these measures address upstream determinants of postprandial lipemia and confer cardiometabolic benefits beyond lipid metabolism alone [8,11,79].

Pharmacologic interventions are most relevant in patients with persistently elevated TG, high cardiovascular risk, or metabolic conditions in which lifestyle modification alone is insufficient. Long-chain omega-3 fatty acids at adequate therapeutic doses reduce circulating TG concentrations and, in selected populations, have been associated with reductions in cardiovascular events, although responses vary according to baseline TG levels and formulation characteristics [8,80,81,82]. EPA-dominant preparations have generally demonstrated more consistent TG-lowering and cardiometabolic effects than mixed EPA/DHA formulations, particularly in individuals with elevated baseline TGs.

Fibrate therapy improves TG clearance and remnant lipoprotein metabolism, particularly in individuals with combined dyslipidemia characterized by elevated TG and low high-density lipoprotein cholesterol. These effects may be partly mediated through enhanced remnant lipoprotein clearance, making fibrates particularly relevant in hypertriglyceridemic phenotypes characterized by increased remnant cholesterol exposure [80,81,82,83]. Emerging lipid-modifying agents targeting pathways involved in TRL regulation, such as apolipoprotein C-III or angiopoietin-like protein signaling, show promise, although evidence regarding their specific impact on postprandial TG dynamics remains limited [84,85,86,87]. Pharmacologic therapy should therefore be viewed as reinforcing, rather than replacing, dietary optimization.

A pragmatic clinical strategy emphasizes lifestyle and nutritional interventions as first-line measures, with pharmacologic therapy introduced when TG levels remain elevated, pancreatitis risk is a concern, or overall cardiovascular risk justifies intensification. The goal is not only to reduce fasting TG concentrations but also to improve lipid handling and metabolic flexibility across the day, thereby limiting cumulative exposure to TG-rich remnant particles [19,79,88].

The principal nutritional and pharmacological interventions influencing postprandial TG responses are summarized in Table 2 (based on evidence from randomized controlled trials, observational studies, and consensus statements cited in the text).

## 8. Discussion and Clinical Implications

The evidence reviewed in this manuscript highlights postprandial TG metabolism as a dynamic and clinically meaningful dimension of lipid physiology that complements, rather than replaces, traditional fasting lipid assessment. While fasting LDL-C remains a central and causally established target in cardiovascular prevention, postprandial TG responses provide additional insight into metabolic flexibility, dietary exposure, and the efficiency of TRL clearance across the day. The convergence of physiological, epidemiological, and nutritional evidence suggests that repeated daily excursions in TG-rich particles may contribute to a cumulative vascular burden, particularly in contemporary dietary environments characterized by frequent energy-dense meals and extended eating windows. However, this concept is supported primarily by indirect and mechanistic evidence, while direct longitudinal confirmation remains limited. Recent reviews continue to emphasize the growing interest in postprandial TG metabolism as an integrative marker linking nutrition, metabolic flexibility, and cardiometabolic risk [89]. Part of this burden is likely mediated by variability in remnant lipoprotein exposure, which more closely reflects the atherogenic component of postprandial lipid metabolism than TG concentrations alone.

Within the context of existing literature, the present review extends prior work by integrating mechanistic, nutritional, and clinical perspectives on postprandial TG metabolism. By framing postprandial TG dynamics as a functional marker of metabolic flexibility and residual cardiovascular risk, this synthesis aims to bridge experimental evidence with clinically relevant interpretation and support a more comprehensive understanding of lipid-related cardiometabolic risk.

A consistent finding across studies is the substantial inter-individual variability of postprandial TG handling. This variability appears closely related to insulin sensitivity, body fat distribution, hepatic lipid metabolism, and habitual dietary patterns. Individuals with insulin resistance, type 2 diabetes, obesity, and MASLD repeatedly demonstrate exaggerated or prolonged postprandial lipemia compared with metabolically healthy controls. In these populations, fasting TG concentrations alone may underestimate exposure to cholesterol-enriched remnant lipoproteins and fail to capture disturbances that are primarily expressed in the fed state. Conversely, metabolically healthy individuals typically exhibit efficient and transient TG clearance, underscoring that postprandial lipemia becomes clinically relevant primarily when clearance mechanisms are impaired.

Dietary composition and eating behavior emerge as central and modifiable determinants of postprandial TG dynamics. Evidence from controlled feeding studies and observational research indicates that meal size, fat quality, refined carbohydrate intake, fructose exposure, alcohol consumption, and overall dietary patterns influence both the amplitude and duration of TG excursions. These effects interact with the underlying metabolic phenotype, such that the same dietary stimulus may elicit modest responses in some individuals and exaggerated excursions in others. This perspective supports the prioritization of dietary and lifestyle interventions as foundational strategies for improving postprandial lipid handling. Postprandial TG metabolism also appears acutely responsive to physical activity, with even a single session of moderate-intensity aerobic exercise capable of attenuating next-meal TG excursions. Emerging evidence also suggests a potential role of gut microbiota and bile acid signaling in modulating postprandial lipid metabolism, although these mechanisms remain incompletely understood and currently have limited direct clinical applicability [90,91].

From a clinical perspective, TG evaluation should be interpreted within a broader metabolic context rather than as a single fasting laboratory value. While fasting lipid panels remain essential for cardiovascular risk stratification and therapeutic decision-making, exclusive reliance on fasting TGs may overlook disturbances in lipid handling that occur throughout the day and create discordance between apparent lipid control and actual lipemic exposure. Incorporating non-fasting measurements or contextual postprandial assessment, particularly in individuals with metabolic vulnerability, may therefore provide complementary insight into residual cardiometabolic risk without altering established lipid management frameworks.

Certain patient groups appear particularly likely to benefit from greater attention to postprandial TG dynamics. Individuals with insulin resistance, type 2 diabetes, obesity, metabolic syndrome, and MASLD frequently demonstrate prolonged or exaggerated TG excursions and increased remnant lipoprotein exposure that may not be evident in fasting profiles. Additional consideration may also be warranted in women with PCOS and in older adults experiencing age-related declines in metabolic flexibility. In these contexts, postprandial TG assessment should be viewed as an adjunctive tool capable of refining risk perception and supporting lifestyle-oriented interventions rather than as a standalone diagnostic test.

Despite increasing interest, several areas of uncertainty and methodological heterogeneity limit direct translation into standardized clinical practice. Study protocols vary substantially with respect to meal composition, sampling intervals, duration of observation, and outcome definitions, making cross-study comparisons difficult. No universally accepted threshold currently defines an abnormal postprandial TG response, and consensus is lacking regarding optimal timing for measurement outside research settings. Much of the available evidence derives from short-term interventions or observational studies, which constrain causal inference and long-term risk prediction. These limitations have important implications for the interpretation of the present findings, as they preclude definitive clinical recommendations and limit the ability to establish standardized thresholds. Accordingly, the conclusions of this review should be interpreted as integrative and hypothesis-generating rather than prescriptive, highlighting the need for further prospective and interventional studies.

Selective rather than universal clinical application therefore appears most defensible. Non-fasting TG measurements are practical for routine screening and may better reflect daily metabolic exposure, whereas targeted postprandial evaluation appears most informative in individuals with metabolic vulnerability or discordance between clinical risk and fasting lipid values. Postprandial data should function as supportive information rather than as a standalone diagnostic criterion. Lifestyle optimization, including dietary quality, weight management, and regular physical activity, remains the primary intervention, with pharmacologic therapies serving as adjuncts in patients with persistent hypertriglyceridemia or elevated cardiovascular risk.

### Future Directions

Future research should prioritize standardization and clinical applicability. Well-designed prospective studies are needed to identify which postprandial TG patterns, such as prolonged time-to-baseline or increased area-under-the-curve, most strongly predict long-term cardiovascular and metabolic outcomes and to establish pragmatic thresholds suitable for clinical use. Greater emphasis on diverse populations, real-world dietary patterns, and longitudinal follow-up will be essential for improving generalizability.

In parallel, research integrating nutritional interventions, circadian biology, digital metabolic monitoring, and emerging lipid-modifying therapies may help define how dynamic TG responses can be incorporated into personalized prevention strategies. Advancing methodological consistency and clinical applicability will be essential for determining whether postprandial TG assessment evolves from a predominantly research-oriented construct into a structured component of routine cardiometabolic care focused on metabolic flexibility and residual cardiovascular risk.

## 9. Conclusions

Postprandial TG metabolism represents a dynamic and clinically informative dimension of lipid physiology that extends beyond the information provided by fasting lipid panels alone. While LDL-C remains the primary therapeutic target in cardiovascular prevention, accumulating evidence indicates that TRLs and their cholesterol-enriched remnants contribute to residual cardiometabolic burden despite apparently adequate fasting lipid control. Because the postprandial state occupies a substantial portion of daily life, postprandial TG responses provide a relevant window into metabolic flexibility and the efficiency of lipid clearance across the day.

Dietary composition, eating patterns, and overall lifestyle behaviors are central determinants of postprandial TG responses, highlighting the pivotal role of nutrition in both assessment and management. Individuals with insulin resistance, type 2 diabetes, obesity, and MASLD appear particularly susceptible to exaggerated or prolonged postprandial lipemia, even when fasting TG values are not markedly elevated. In these settings, non-fasting measurements and contextual interpretation of TG dynamics may provide complementary insight into cardiometabolic risk when integrated into broader clinical evaluation.

Although the absence of standardized protocols and universally accepted thresholds currently limits routine implementation, a pragmatic approach emphasizing dietary quality, weight management, regular physical activity, and adjunctive pharmacologic therapy when indicated may support improved TG handling and metabolic flexibility. Until clearer consensus emerges, postprandial TGs should be viewed not as a replacement for established lipid markers, but as a complementary perspective that refines the understanding of residual cardiovascular risk and supports a more comprehensive approach to cardiometabolic assessment.

At the same time, the current evidence base remains heterogeneous and largely derived from observational and short-term studies, limiting causal inference and immediate clinical standardization. Therefore, postprandial TG assessment should currently be regarded as a complementary and evolving tool, with its integration into routine practice requiring further prospective validation and consensus development.

## Figures and Tables

**Table 1:** Clinical contexts associated with altered postprandial TG responses and targeted nutritional strategies (conceptual summary).

Clinical Context	Typical Postprandial TG Pattern	Contributing Mechanisms (General)	Clinical Implication	Targeted Nutritional Strategies
Type 2 diabetes/Insulin resistance	Higher peaks, delayed return to baseline	Increased VLDL secretion, reduced LPL activity, impaired remnant clearance	Fasting TG may underestimate cumulative lipemic burden [4,7,28]	Reduce refined carbohydrates and fructose; emphasize low-GI carbohydrates; replace saturated fats with unsaturated fats
Obesity/Metabolic syndrome	Prolonged elevation, increased remnant particles	Visceral adiposity, elevated free fatty acid flux, chronic low-grade inflammation	Postprandial assessment may refine cardiometabolic risk beyond static lipid panels [70,71,72]	Caloric moderation; Mediterranean-style dietary pattern; increased fiber intake
MASLD	Sustained elevation despite variable fasting TG	Hepatic steatosis, increased de novo lipogenesis, altered VLDL production	Liver status is relevant for lipid interpretation and residual risk estimation [73,74,75]	Reduce fructose and ultra-processed foods; Mediterranean-style dietary pattern
PCOS	Moderate–high excursions in insulin-resistant phenotypes	Hormonal dysregulation, insulin resistance, altered adipokine signaling	Consider broader metabolic screening beyond fasting lipids [76]	Low-glycemic-index dietary pattern; balanced macronutrient distribution; weight management
Aging	Gradual prolongation of clearance and greater inter-individual variability	Reduced insulin sensitivity, changes in body composition, diminished lipolytic efficiency	Dynamic lipid handling may change even in the absence of overt disease [29,77]	Smaller meal size; attention to meal timing; balanced macronutrient intake

Nutritional strategies are derived from the body of evidence discussed in the text.

**Table 2:** Nutritional and pharmacological interventions influencing postprandial TG responses.

Intervention Domain	Examples	General Effect on Postprandial TG	Additional Clinical Considerations
Dietary pattern & composition	Unsaturated fats, high-fiber foods, reduced refined carbohydrates	Reduced TG peak and improved clearance	Broad cardiometabolic benefits; first-line approach [17,21,53]
Weight management	Caloric moderation, sustained weight loss	Reduced amplitude and duration	Improves insulin sensitivity and hepatic lipid metabolism [70,71,72]
Physical activity	Regular aerobic and resistance exercise	Improved TG clearance	Effects observed even with short-term activity [23]
Meal structure	Structured meals, reduced snacking	Reduced cumulative exposure	Supports metabolic recovery between meals [56,57]
Omega-3 fatty acids	Prescription or high-dose omega-3 formulations	Reduced TG concentrations	Response varies with baseline TGs and formulation [82,83]
Fibrates	Fenofibrate, gemfibrozil	Improved TG clearance	Consider in combined dyslipidemia; monitor interactions [79,80]
Emerging therapies	Agents targeting TRL pathways	Potential TG reduction	Long-term and postprandial data still evolving [84,85,86,87]

## Data Availability

No new data were created or analyzed in this study. Data sharing is not applicable to this article.

## Abbreviations

The following abbreviations are used in this manuscript:

**Abbreviation**	**Definition**
ASCVD	Atherosclerotic Cardiovascular Disease
AUC	Area Under the Curve
DHA	Docosahexaenoic Acid
EPA	Eicosapentaenoic Acid
HDL-C	High-Density Lipoprotein Cholesterol
LDL-C	Low-Density Lipoprotein Cholesterol
LPL	Lipoprotein Lipase
MASLD	Metabolic-Associated Steatotic Liver Disease
PCOS	Polycystic Ovary Syndrome
RCT	Randomized Controlled Trial
TG	Triglyceride
TRLs	Triglyceride-Rich Lipoproteins
VLDL	Very-Low-Density Lipoprotein
